# Gene–Environment Interaction Mendelian Randomization Reveals Statin-Dependent Associations Between Metabolites and Lipid Traits

**DOI:** 10.3390/ph19091475

**Published:** 2026-09-17

**Authors:** Aleem Razzaq, Najeha Anwardeen, Khaled Naja, Asma A. Elashi, Gaurav Thareja, Ilhame Diboun, Karsten Suhre, Mohamed A. Elrayess

**Affiliations:** 1 Biomedical Research Center, QU Health, Qatar University, Doha P.O. Box 2713, Qatar; aleem.razzaq@qu.edu.qa (A.R.); n.anwardeen@qu.edu.qa (N.A.); khaled.naja@qu.edu.qa (K.N.); asma.elashi@qu.edu.qa (A.A.E.); 2 MRC Centre for Neurodevelopmental Disorders, King’s College London, London P.O. Box SE1 1UL, UK; gat2010@qatar-med.cornell.edu; 3 Medical and Population Genomics Lab, Sidra Medicine, Doha P.O. Box 26999, Qatar; idiboun@sidra.org; 4 Bioinformatics Core, Weill Cornell Medicine-Qatar, Education City, Doha P.O. Box 24144, Qatar; kas2049@qatar-med.cornell.edu; 5 Englander Institute for Precision Medicine, Weill Cornell Medicine, New York, NY 10065, USA; 6 College of Medicine, QU Health, Qatar University, Doha P.O. Box 2713, Qatar

**Keywords:** Mendelian randomization, gene–environment interaction, metabolomics, lipid traits, statins, polygenic risk score

## Abstract

**Introduction/Background**: Observational links between circulating metabolites and lipid traits are often confounded by environmental factors. This study applied Mendelian randomization (MR) and gene–environment interaction MR (MR-G×E) to assess the average causal effects of genetically predicted metabolites on lipid traits and their modification by statin use. **Methods**: Participants (*n* = 2810) from Qatar Biobank were analyzed including genetics, metabolomics and lipid profiles. The cohort was divided into discovery (*n* = 1968) and validation (*n* = 842). One-sample MR using two-stage least squares regression was applied to estimate the average causal effect of metabolites on LDL, HDL, and Triglycerides, while MR-G×E analysis was also applied to investigate whether genetically predicted metabolite–LDL/HDL associations differ by statin use. Genetic variants associated with metabolites were used to generate a polygenic risk score (PRS). The interaction term between the PRS and statin use was incorporated in MR-G×E, while adjusting for confounders. **Results**: A total of 167 metabolites were commonly associated with LDL, HDL, and Triglycerides. One-sample MR showed no significant average causal effect of genetically predicted metabolites on lipid traits in the overall population. However, MR-G×E analysis revealed significant statin-dependent effects. Genetically predicted 2-amino-octanoate and cysteine–glutathione disulfide demonstrated strong and reproducible positive interaction effects with LDL levels in statin users in both discovery and validation cohorts. While no overall average causal effects were observed, MR-G×E revealed that genetically predicted metabolites can influence LDL levels in the context of statin use. **Conclusions:** These findings highlight the importance of incorporating pharmacological exposures in MR analyses and support the role of drug–metabolite interactions in precision lipid management.

## 1. Introduction

In recent years, metabolomics has emerged as an important technique to identify metabolites, which depict the biochemical state of an organism. With the emergence of approaches like mass spectrometry (MS) and nuclear magnetic resonance (NMR) chromatography, metabolomics serves as a tool to investigate the metabolic profiles of an individual in different disease conditions [1,2]. Since circulating metabolites play an important role in understanding the metabolism of lipids, energy homeostasis, and oxidative pathways, metabolomics serves as a useful approach to investigate the behavior of clinical traits like Triglycerides, low-density lipoprotein (LDL), and high-density lipoprotein (HDL) in cardiovascular diseases [3,4]. Multiple studies have identified metabolites associated with lipid traits; however, these associations were mostly observed in either cross sectional or observational studies, which are difficult to interpret because they may be influenced by external factors, dietary factors, and medication use [5,6].

Statins are among the most important and widely used drugs in cardiovascular diseases. They lower the blood total cholesterol and LDL cholesterol by inhibiting the 3-hydroxy-3-methylglutaryl-coenzyme A (HMG-CoA) reductase [7,8]. However, due to host genetic susceptibility and external environment interactions, the lipid lowering effects of statins are heterogenous, and can alter different metabolic pathways, thus potentially altering the circulating metabolic profiles [9,10]. This indicates that the effect of some metabolites could become more apparent in the presence of pharmacological exposure, which can behave as an effect modifier in the metabolomics studies [11]. Understanding how statin use modifies metabolite–lipid relationships is therefore essential for accurately interpreting statin-dependent genetic interaction effects and may help in the identification of metabolic pathways that represent context-specific therapeutic targets.

Genetic variation has enabled the investigation of metabolite-associated variants, which can serve as an instrument variable (IV) in a Mendelian randomization (MR) framework. This approach helps to investigate whether genetically predicted metabolites could affect lipid traits, thus minimizing the bias arising from measured and unmeasured confounding factors [3]. Conventional MR methods like two-stage least square regression (2SLSR) ‘ivreg’ method estimate the average causal effect across populations; however, this method does not account for heterogeneity and horizontal pleiotropy arising from environmental and pharmacological exposures [12,13]. To address this limitation, gene–environment interaction Mendelian randomization (MR-G×E), a modified version of 2SLSR, was developed [14]. However, this approach has not been used extensively in the context of drug–metabolite interactions.

In this study, using one-sample MR and MR-G×E framework, we integrated genetics, metabolomics, and clinical data from a large Qatar Biobank (QBB) cohort to investigate the associations of genetically predicted metabolites with lipid traits and whether these associations are modified by statin use. This integrative framework offers insight into statin-dependent genetic interaction effects and the interplay between metabolism, genetics, and pharmacological exposure within a causal-inference framework.

## 2. Results

### 2.1. Metabolites Associated with LDL, HDL, and Triglycerides

**Figure 1:**
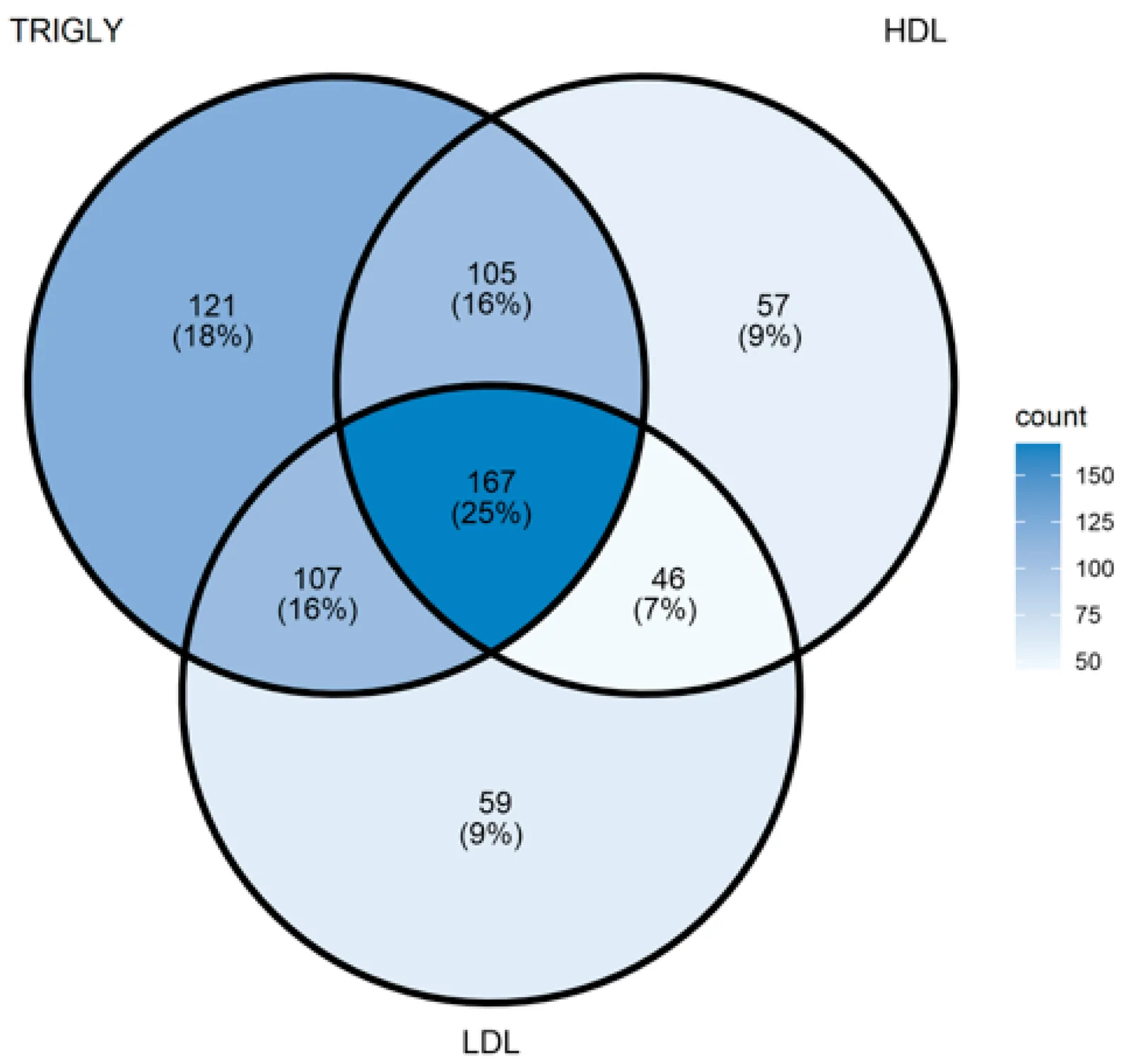
Metabolites associated with HDL, LDL, and Triglycerides.

In the current study, the association between metabolites and LDL, HDL, and Triglycerides was investigated, while adjusting for age, gender, BMI, fasting time, statin use, and PC1–2. Overall, 379 metabolites showed significant (FDR adj *p* < 0.05) association with LDL, 375 metabolites showed significant association with HDL, and 500 metabolites showed significant association with Triglycerides. Among the metabolites significantly associated with the three lipid traits, 121 were uniquely associated with Triglycerides, 57 with HDL, and 59 with LDL. In addition, 105 metabolites were shared between Triglycerides and HDL, 107 between Triglycerides and LDL, and 46 between HDL and LDL, while 167 metabolites were common to all three lipid traits (Figure 1). The list of metabolites associated with each lipid panel can be found in Supplementary Tables S1–S4.

### 2.2. Mendelian Randomization Analysis

In this study, the average causal effect of genetically predicted metabolites on HDL, LDL, and Triglycerides levels were investigated in an overall population, irrespective of statin use, using one-sample MR. In addition, the MR-G×E interaction was applied to investigate whether the association between genetically predicted metabolites and HDL, LDL, and Triglyceride levels differed by statin use.

For this purpose, SNPs associated (5 × 10^−6^) with each of the common metabolites of lipid panels were used to generate a PRS, which served as IVs (Supplementary Table S5). Most of the IVs (PRS) were unable to pass the necessary MR assumptions in the discovery phase, as they showed significant (*p* < 0.05) association with either outcomes (HDL/LDL/Triglycerides) or statin use which can introduce a horizontal pleiotropic effect or collider bias.

In the discovery cohort, only metabolites like glutamate, 2-amino-octanoate, cysteine–glutathione disulfide, and gama-glutamyl glycine associated with LDL and HDL passed the necessary MR assumptions. F-statistics were calculated to check the strength of each IV (PRS) (Supplementary Table S6/Supplementary Figure S1). In this study, no significant results were observed for Triglycerides; therefore, the current study focused only on LDL and HDL for further MR analysis.

#### 2.2.1. MR Analysis of LDL

In the discovery cohort, no evidence of an average causal effect of genetically predicted 2-amino-octanoate (β = −0.004, *p* = 0.93), cysteine–glutathione disulfide (β = 0.0112, *p* = 0.864), and glutamate (β = 0.10, *p* = 0.38) on LDL levels was observed, irrespective of statin use, while adjusting for covariates (age, gender, BMI, and ancestral PC1–3). The same results were also confirmed in the validation cohort (Supplementary Table S7/Supplementary Figure S2).

**Figure 2:**
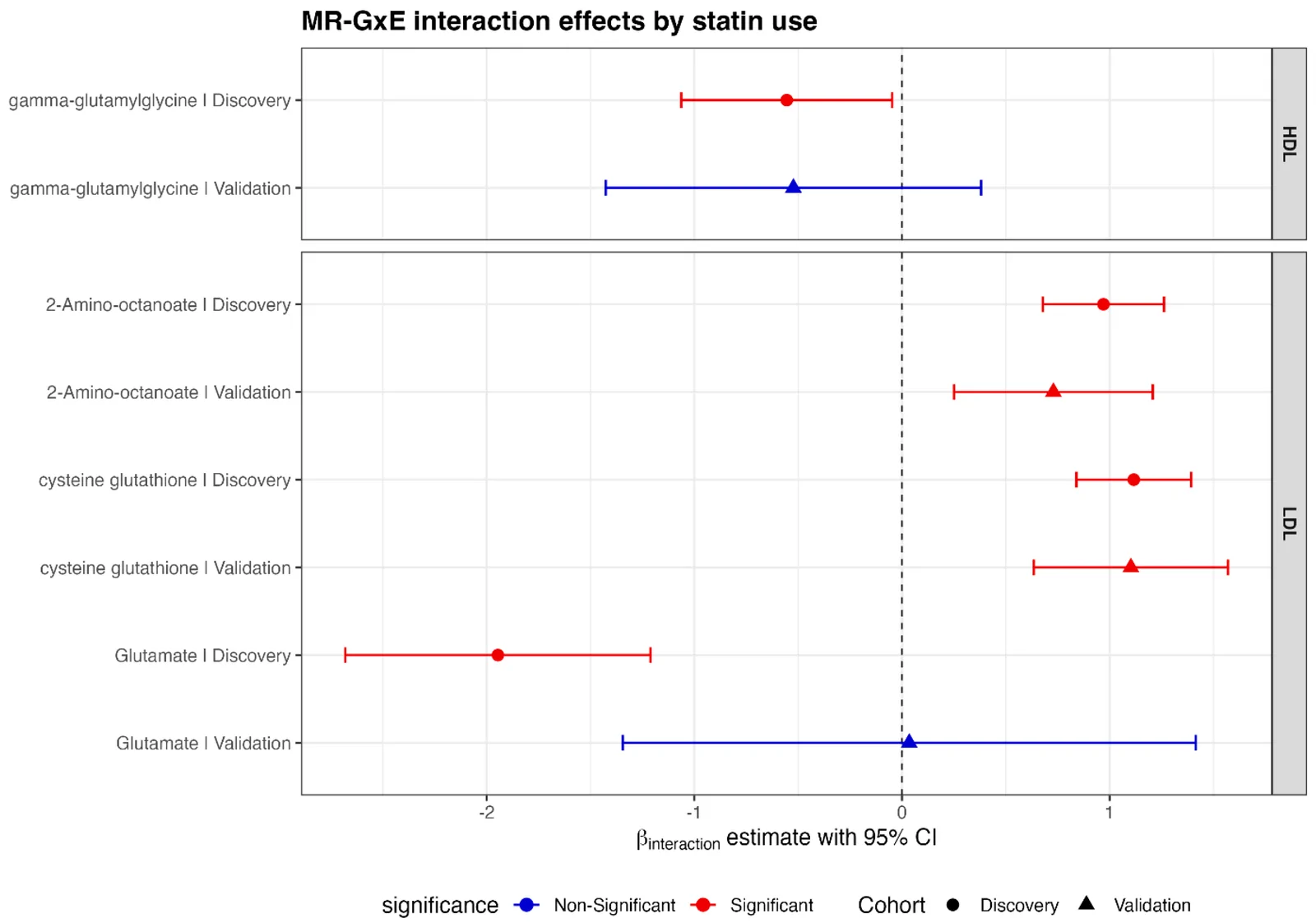
MR gene–environment interaction in statin users: Forest plot represents the β-interaction estimates (95% CI) of genetically predicted metabolites on HDL and LDL levels in discovery and validation cohort. Red points represent the significant interaction effect while blue points represent the non-significant interaction effect. 2-amino-octanoate and cysteine–glutathione disulfide showed statin-dependent significant interaction with LDL. Gama-glutamyl-glycine and glutamate showed significant interaction in HDL and LDL respectively in the discovery cohort; however, the interaction effect was not replicated in the validation cohort.

The MR-G×E analysis in the discovery cohort (Table 1) showed significant statin-dependent interaction between genetically predicted 2-amino-octanoate (PRS) and LDL levels in statin users (β_interaction = 0.97, *p* = 9.04 × 10^−11^) when compared with non-users. The positive β_interaction showed the association between the 2-amino-octanoate-associated PRS and LDL levels was more positive among statin users than non-users (Reference group). This interaction effect was replicated in the internal validation cohort with a consistent direction effect (β_interaction = 0.72, *p* = 0.0029) and significance (Figure 2). Similarly, MR-G×E analysis in the discovery cohort revealed significant statin-dependent interaction between genetically predicted cysteine–glutathione disulfide (PRS) and LDL levels in statin users (β_interaction = 1.116, *p* = 3.96 × 10^−15^) when compared with non-users. This interaction effect was replicated in the internal validation cohort with a consistent direction effect (β_interaction = 1.102, *p* = 4.47 × 10^−6^) and significance. This β_interaction estimate showed higher levels of cysteine–glutathione disulfide-associated PRS were associated with higher levels of LDL in statin users when compared with non-users.

#### 2.2.2. MR Analysis in HDL

In the case of HDL, one-sample MR analysis (Table 2) showed no evidence of an average causal effect of genetically predicted gama-glutamyl glycine on HDL levels in an overall population (β = −0.011, *p* = 0.912), irrespective of statin use. The MR-G×E analysis in the discovery cohort revealed a significant statin-dependent interaction between genetically predicted gama-glutamyl glycine and HDL levels in statin users (β_interaction = −0.55, *p* = 3.22 × 10^−2^). The β_interaction effect showed higher levels of gama-glutamyl glycine-associated PRS were associated with low levels of HDL in statin users relative to non-users (Reference group). However, this significant interaction was not replicated in the validation cohort (β_interaction = −0.523, *p* = 0.257), which showed the observed interaction effect may not be robust.

#### 2.2.3. Sensitivity Analysis

Leave-one-out sensitivity analysis was performed to assess the robustness of observed results and the horizontal pleiotropic effect. In the case of glutamate, MR G×E analysis in the discovery cohort revealed a significant statin-dependent interaction between genetically predicted glutamate and LDL levels in statin users (β_interaction = −1.95, *p* = 2.38 × 10^−7^). Although this negative interaction estimate was significant in the discovery cohort, this interaction effect was weaker and non-significant in the internal validation cohort (β_interaction = −1.01, *p* = 0.062). Furthermore, this weaker interaction effect in the validation cohort was further lost after performing sensitivity analysis as the removal of a single SNP abolished the effect (β = 0.03, *p* = 0.96) (Supplementary Table S7). In contrast, the interaction effect of 2-amino-octanoate, cysteine–glutathione disulfide, and gama-glutamyl glycine remained consistent even after the sensitivity analysis, showing the robustness of the observed results. There was no evidence of heterogeneity between the discovery and internal validation interaction estimates for either 2-amino-octanoate (*p* difference = 0.399) or cysteine–glutathione disulfide (*p* difference = 0.959), indicating consistency of the interaction estimates across the two cohorts.

## 3. Discussion

In this study, we applied one-sample MR along with the gene–environment interaction MR (MR-G×E) to investigate the association of genetically predicted metabolites on lipid traits and whether this association is modified by statin use. In an overall population, although no evidence of an average causal effect of genetically predicted metabolites on lipid traits was observed, the study observed evidence that a genetically predicted metabolite effect may modify the LDL levels in statin users.

The non-significant average causal effect of metabolites in an overall population, and significant MR-G×E interaction effects in statin users, suggests the relationship between metabolites and lipid traits does not extend population-wide; however, it could be conditionally modified by statin exposure. This is consistent with the previous findings, which suggested that the observed metabolite–lipid association in observational data does not appear to be causal when used as a genetic instrument [15]. However, the MR-G×E interaction showed that these null effects can mask the statin-dependent relationship between genetically predicted metabolites and lipid levels.

Notably, in the discovery and validation cohort, 2-amino-octanoate and cysteine–glutathione disulfide-associated PRS showed strong and reproducible interaction effects with LDL in statin users. The interaction effect (β_interaction) showed that higher levels of genetically predicted (PRS) metabolites are associated with increased LDL levels in statin users. This showed the influence of pharmacological exposure when investigating the effect of metabolites on LDL levels.

There is very limited literature on 2-amino-octanoate, which is derived from food or produced via the host and gut microbial metabolism of dietary components. Van der Spek et al. [16] showed that individuals with depression had lower plasma levels of 2-amino-octanoate compared with non-depressed participants. Interestingly, depression was shown to be strongly related to LDL [17], supporting the notion that 2-amino-octanoate lies on a shared lipid–mood metabolic axis. In a recent study [18], N-acetyl-2-amino-octanoate, the acetylated form of 2-amino-octanoate, was identified among the metabolites with statistically robust signals for cardiovascular traits such as atrial fibrillation, where genetically higher metabolite levels are associated with higher disease risk. The observed interaction result in this study suggests that the relationship between LDL and 2-amino-ocatanoate could become more apparent when studied in an altered metabolic environment induced by statin therapy. Since statins inhibit HMG-CoA reductase and thereby influence downstream pathways involved in fatty acid, cholesterol, and amino acid metabolism, the metabolic context in statin users is likely distinct from that of non-users. In this altered environment, genetically higher 2-amino-octanoate levels may contribute to, or reflect, differential LDL remodeling and clearance under statin exposure. Accordingly, the observed MR results may be better explained by statin-dependent metabolic effects that conditionally modify the LDL–2-amino-octanoate relationship, rather than by a single population-wide association that is constant across treated and untreated individuals.

Cysteine–glutathione disulfide is part of the glutathione redox system that regulates cellular thiol–disulfide balance. Interestingly, statins may increase or preserve antioxidant capacity, which could shift cysteine/glutathione redox balance [19]. A recent study by Martínez-Gili et al. [20] suggests that cysteine–glutathione disulfide may have a protective role against early hypercholesterolemia-induced liver injury, likely by restoring antioxidant defenses and mitigating oxidative stress. The association between cysteine–glutathione disulfide and LDL observed only among statin users may reflect a statin-specific interaction between lipid reduction and redox regulation, where LDL levels and thiol–disulfide balance become coupled only after pharmacologic perturbation of cholesterol metabolism and oxidative pathways.

Clinically, our study frames these two novel metabolites as candidate markers of statin resistance or suboptimal LDL response, analogous to how variants in APOE, LPA, and CETP modulate statin-related lipoprotein profiles even when total LDL appears to be similar [21]. Such markers could help identify patients who might benefit from earlier use of combination therapy rather than statin monotherapy. However, this should be regarded as largely speculative at this stage and would require replication in larger independent cohorts and detailed mechanistic studies before translation into clinical practice. Future work integrating longitudinal metabolomics, more statin exposure data, and experimental models will be essential to determine whether these metabolites are associated with variability in statin response and to further investigate their potential role in LDL reduction.

The MR-G×E interaction in glutamate results indicates an important nuance. Although the discovery cohort showed a significant interaction between genetically predicted glutamate and LDL levels in statin users, this interaction effect was not consistent in the validation cohort. The significance was lost due to the removal of a single SNP as a result of sensitivity analysis. These results suggest that the interaction effect was unstable and driven by a single genetic variant (rs11449874), which also showed the fragility of the instrument variables. Such results are very challenging to process in one-sample MR and MR-G×E environments, where weak instruments could produce strong but inflated or non-reproducible findings [22].

A similar pattern was observed for gama-glutamyl glycine, where the interaction results with HDL were not replicated in the validation cohort. In the discovery cohort, gama-glutamyl glycine showed a significant inverse interaction effect in statin users, suggesting that increased levels of genetically predicted gama-glutamyl glycine are associated with low levels of HDL; however, in the validation cohort, the results were not validated. Since the HDL metabolism is influenced by multiple pathways like apolipoprotein A-I (ApoA-I synthesis), reverse cholesterol transport, and cholesteryl ester transfer protein (CETP)-mediated lipid exchange, the effect of a single metabolite on HDL levels will be modest, especially if the strength of the instrument is weak.

A major strength of this study is the implementation of MR along with MR-G×E, which leverages the integration of genetics, metabolomics, and pharmacological exposures, and captures statin-dependent interaction inferences. Moreover, the use of discovery and validation cohorts helps to strengthen the robustness of the findings. The strict filtering of SNPs and the use of a PRS as an instrument variable minimize the bias in identifying weak instruments. However, there are certain limitations to the study which should be also considered. Firstly, the relatively small number of statin users in the discovery (*n* = 196) and validation (*n* = 83) cohorts may have reduced the statistical power of the MR-G×E analyses, particularly for detecting small-to-moderate interaction effects. This may partly explain the lack of statistical significance observed for gamma-glutamyl glycine and glutamate in the validation cohort despite the interaction estimates observed in the discovery group. Therefore, non-significant interaction results should be interpreted cautiously and should not be considered evidence for the absence of statin-dependent interaction effects. Furthermore, the relatively small statin-treated subgroup may limit the precision and generalizability of the findings, highlighting the need for replication in larger independent cohorts with greater numbers of statin users. Secondly, the study was performed using the Qatar Biobank data only, and did not replicate the findings in other populations, thus limiting the generalizability of the findings. Thirdly, statin use in this study was treated as a binary exposure and does not take into account the duration, dosage, and type of statins used, which may influence the metabolic effect. Also, single-time-point metabolomics data was used in this study, which cannot assess variation due to external factors like medication, diet, and physical activity.

## 4. Methods

### 4.1. Study Cohort

Study participants were enrolled from the Qatar Biobank (QBB), and informed consent was taken from each participant before collecting the samples and associated clinical data [23]. The enrolled participants were asked to complete a standardized questionnaire related to medical history, diet, and medication intake. This study comprised 2810 participants with available genomics, metabolomics and clinical data, including 279 statin users. To perform the analysis, the study participants were divided into discovery (*n* = 1968; 70%) and internal validation (*n* = 842; 30%) cohorts, using a stratified random split based on statin use. The discovery subset included 196 statin users and 1772 non-users, whereas the internal validation subset included 83 statin users and 759 non-users. Table 3 shows that related individuals were excluded during previously described genetic quality control procedures [24]; therefore, no known familial overlap was present between the discovery and internal validation cohort.

### 4.2. Whole-Genome Sequencing of QBB Participants

Whole-genome sequencing (WGS) of QBB participants was performed at the Qatar Precision Health Institute (QPHI) as described previously [25]. Briefly, DNA was extracted from the peripheral blood, and Qiagen MIDI kit (Qiagen, Hilden, Germany) was used to generate genomic libraries. The libraries were sequenced at Sidra Medicine’s facility using Illumina (San Diego, CA, USA) HiSeq X Ten (30×) platform. FastQC (https://www.bioinformatics.babraham.ac.uk/projects/fastqc/, accessed on 18 August 2026) was used to check the quality of fastq files, and bwa.kit (v0.7.12) was used to align reads against human-reference genome GRCh38. Mapping quality was assessed using Picard (v1.117) and gVCF files were generated using GATK 3.4 best practices.

### 4.3. Metabolomics

An untargeted metabolomics approach was used to analyze serum samples from QBB (*n* = 2810) participants using Metabolon platform at the Anti-Doping Lab, Doha, Qatar. The protocol used for the analysis was described in detail previously [26]. Briefly, metabolic profiling was performed on a Waters ACQUITY ultra-performance liquid chromatography (UPLC) system (Waters Corporation, Milford, MA, USA) coupled to a Thermo Scientific Q-Exactive high-resolution accurate-mass spectrometer (Thermo-Fisher Scientific, Inc., Waltham, MA, USA). The instrument was equipped with a heated electrospray ionization (HESI-II) source and an Orbitrap mass analyzer operating at 35,000 mass resolution. Serum proteins were precipitated with methanol, and the resulting extract was split into five fractions to enable broad metabolite coverage using complementary ionization and chromatographic conditions. Raw data extraction, peak detection, and quality control were carried out using Metabolon’s proprietary software (Metabolon Inc., Durham, NC, USA). Metabolites were annotated by matching features to a library of ~3300 authenticated reference standards, and each match was manually reviewed and corrected where required to ensure accurate identification.

### 4.4. Lipid Panel

Metabolomics data was transformed and normalized prior to analysis using bestNormalize package. Associations between circulating lipid markers and individual metabolite levels were assessed using linear regression models. For each metabolite, a separate model was fitted with metabolite intensity as the dependent variable and the lipid marker as the main independent variable. Triglycerides, HDL cholesterol, and LDL cholesterol were evaluated in separate models. Each model was adjusted for BMI, age, sex, fasting status, statin use, and the first two metabolomics-derived principal components. Principal components were generated from the metabolomics dataset using principal component analysis implemented in the mixOmics package. The first two principal components were included as covariates to account for major sources of variation in the metabolomics data. The lipid-marker regression coefficient, standard error, and *p*-value were extracted for each metabolite. Multiple testing correction was applied across metabolites using the false discovery rate method. All analyses were performed using R (v 4.2.1).

### 4.5. Mendelian Randomization and Gene × Environment Interaction Analysis

**Figure 3:**
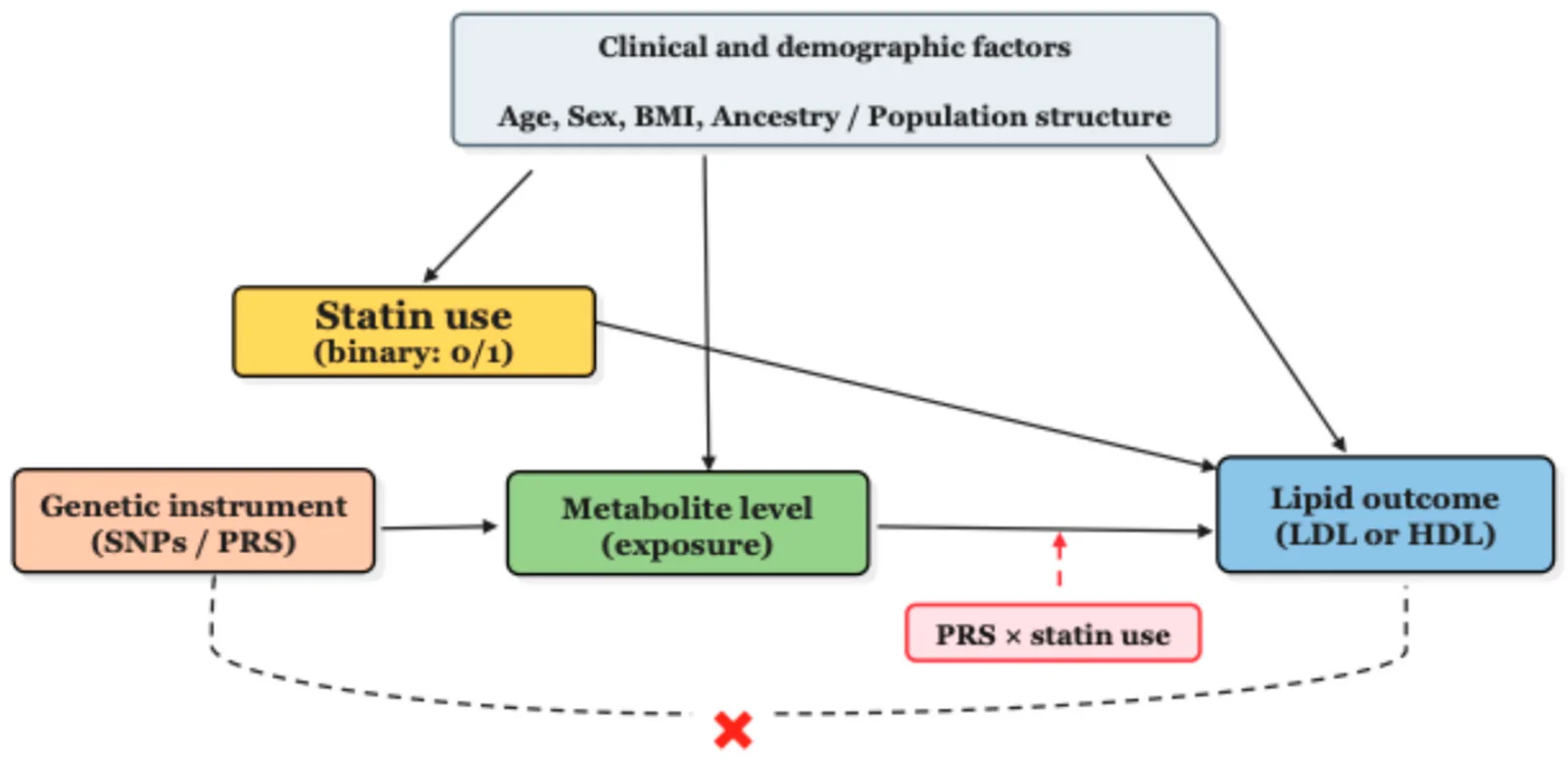
MR-G×E interaction graph: The genetic instrument is associated with metabolite level. The instrument is not associated with the confounders. The instrument affects lipid outcome only through the metabolite. Statin use could be influenced by clinical factors. The red dashed arrow shows the effect modifier. The PRS × statin use term tests whether the genetically predicted metabolite–lipid association differs by statin use. Models adjusted for covariates (age, sex, BMI and ancestral PCs).

In this study, to perform one-sample Mendelian randomization (MR) analysis, the two-stage least square regression (2SLSR) ‘ivreg’ method was applied, as the data was obtained from the same population. Additionally, to perform the gene–environment interaction MR (MR-G×E) analysis, a modified version of the ivreg method was applied as previously published [14,27]. The following formula was used to perform MR-G×E interaction analysis. The product of the PRS and statin users/non-users (binary) was taken as a gene–environment (G × E) interaction variable for the analysis as shown in the formula (Figure 3). To run MR, covariates like age, gender, BMI, and ancestral PCs (PC1–3) were considered.

$$ivreg\left(Outcome\sim Exposure+G\times E+Covariates\;\right|\;PRS+G\times E+Covariates)$$

Here, outcome = lipid traits (LDL, HDL, Triglycerides), Exposure = metabolite values, PRS = PRS associated with metabolites, G × E = PRS × statin use (binary), and Covariates = age, gender, BMI, and ancestral PCs 1, 2, and 3.

#### MR Analysis Parameters

**Figure 4:**
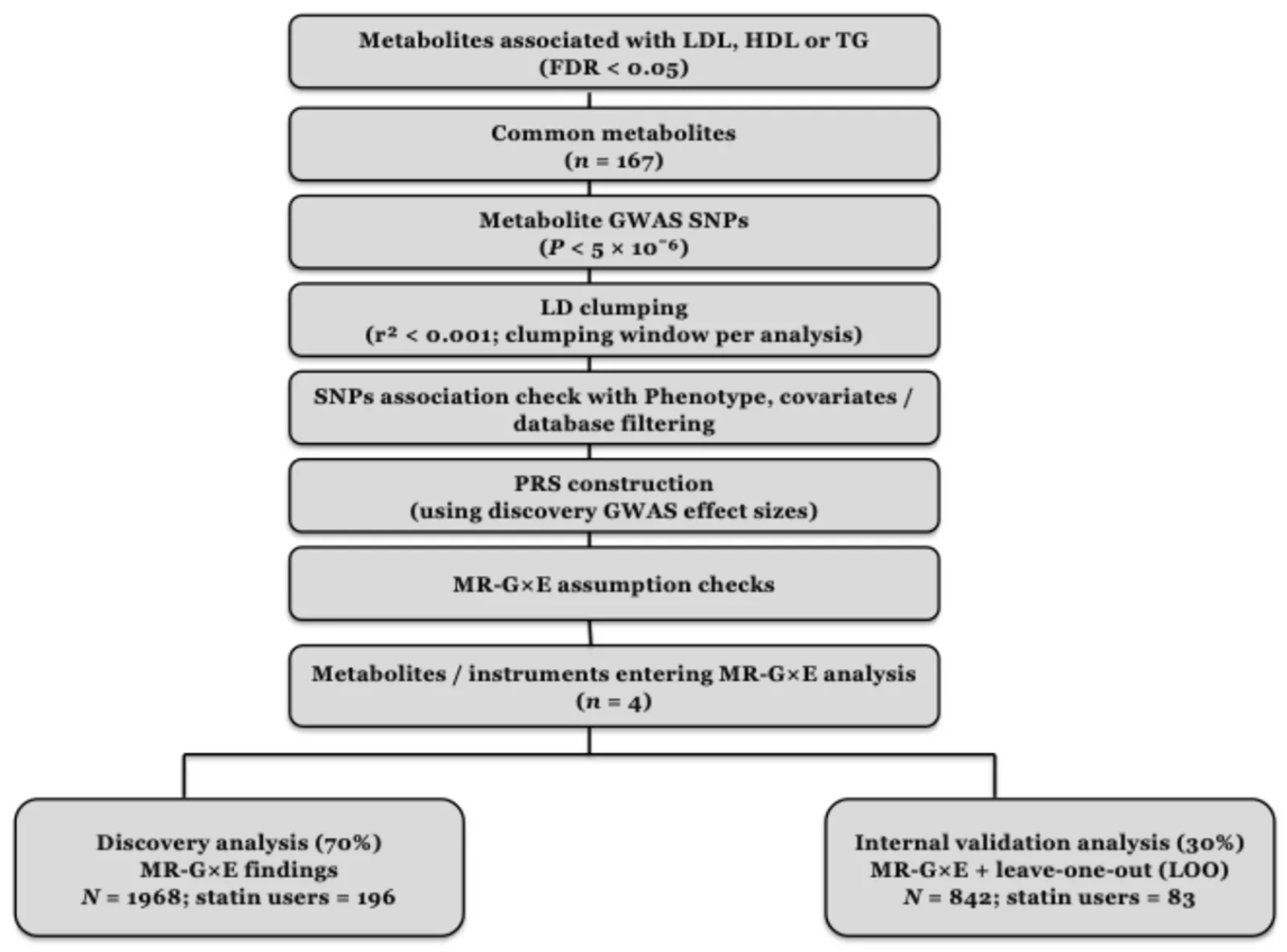
Flow chart for metabolite and instrument selection for MR-G×E.

The common significant metabolites between LDL, HDL, and Triglycerides were identified and were used to perform MR analysis. SNPs associated with these common metabolites were used to calculate the polygenic risk score (PRS) separately. In the discovery cohort, SNPs (*p* < 5 × 10^−6^) associated with each metabolite were clumped using r^2^ = 0.001 within 250 kb window. To reduce the potential influence of horizontal pleiotropy, clumped SNPs were screened for associations (*p* < 0.05) with the lipid outcomes and measured covariates like age, gender, and BMI. Also, to further evaluate horizontal pleiotropy, clumped SNPs were queried in public databases (GWAS catalog and common metabolic diseases knowledge portal: https://hugeamp.org). The variants were screened for previously reported associations with lipid traits. The filtered list of SNPs was used to generate PRSs which will serve as IVs (Figure 4). The PRS was calculated with plink-1.9 using discovery-derived SNP effect estimates as weights [28]. Prior to running the analysis, the MR assumptions were evaluated in both discovery and validation cohorts: (i) the PRS should be strongly associated with the metabolites (exposure). (ii) The PRS should not be directly associated (*p* < 0.05) with the outcome (LDL, HDL, Triglycerides). (iii) The PRS should not be associated (*p* < 0.05) with statin use to avoid collider bias. The PRSs (IV) which failed these assumptions were not considered for MR analysis. The identical SNPs and their corresponding weights derived in the discovery cohort were applied to the internal validation cohort without re-estimation to generate a corresponding PRS. To check the strength of each instrument variable (PRS), F-statistics were calculated using linear regression and IVs with F-statistics > 10 were retained for the analysis. Leave-one-out (LOO) sensitivity analysis was also performed in the discovery cohort as well as in the internal validation cohort using R to check the robustness of the results and horizontal pleiotropy. An MR-G×E interaction identified in the discovery subset was considered internally replicated when the interaction estimate showed the same direction of effect and reached the predefined statistical significance threshold (*p* < 0.05) in the internal validation cohort. Multiple testing in the MR-G×E analysis was controlled using the Benjamini–Hochberg false discovery rate (FDR) method across the four metabolite interaction tests that satisfied the predefined instrumental-variable assumptions and entered the final MR-G×E analysis (Supplementary Table S8). FDR-adjusted *p*-values < 0.05 were considered statistically significant. Consistency of the MR-G×E interaction estimates between the discovery and internal validation cohort was further assessed using a two-sided test for the difference between independent estimates, based on the interaction coefficients and their corresponding standard errors [29]. A *p* difference < 0.05 was considered evidence of a statistically significant difference between the interaction estimates of the two cohort.

## 5. Conclusions

In conclusion, this study showed no average causal effect of genetically predicted metabolites on lipid traits in an overall population. However, MR-G×E analysis revealed genetically predicted metabolite levels could influence LDL/HDL levels in statin users, particularly 2-amino octanoate and cysteine–glutathione disulfide. The findings were confirmed in a validation cohort and highlight the importance of pharmacological exposure in MR studies, supporting wider use of MR-G×E approaches in lipid research and the potential for drug–metabolite interactions to inform future precision lipid-lowering strategies.

## Figures and Tables

**Table 1:** Mendelian randomization gene–environment (MR-G×E) interaction in LDL. The table represents the MR-G×E interaction results observed for LDL in discovery and validation cohorts. In this model, statin use was coded as binary (statin users = 1, non-users = 0), while the statin non-user group served as a Reference group. β_interaction represents the regression coefficient for interaction between the metabolite-associated polygenic risk score (PRS) and statin use (PRS × statin use). Age, gender, BMI, and genetic principal components (PC1–3) are used in the model as covariates. The *p*-values are adjusted using the Benjamini–Hochberg false discovery rate method.

Metabolite	Cohort	β_Interaction	SE	*p*-Adj Value
*2-Amino-octanoate*	Discovery	0.97	0.149	1.81 × 10^−10^
	Validation	0.729	0.244	0.00586
*Cysteine–glutathione disulfide*	Discovery	1.116	0.141	1.58 × 10^−14^
	Validation	1.102	0.239	1.79 × 10^−5^

**Table 2:** Mendelian randomization gene–environment (MR-G×E) interaction in HDL. The table represents the MR-G×E interaction results observed for HDL in discovery and validation cohorts. In this model, statin use was coded as binary (statin users = 1, non-users = 0), while the statin non-user group served as a Reference group. β_interaction represents the regression coefficient for interaction between the metabolite-associated polygenic risk score (PRS) and statin use (PRS × statin use). Age, gender, BMI, and genetic principal components (PC1–3) are used in the model as covariates. The *p*-values are adjusted using the Benjamini–Hochberg false discovery rate method.

Metabolite	Cohort	β_Interaction	SE	*p*-Adj Value
*Gama-glutamine glycine*	Discovery	−0.555	0.259	0.0322
	Validation	−0.523	0.461	0.342

**Table 3:** Study cohort. Total study population: *N* = 2810 (279 statin users and 2531 non-users). Discovery cohort: *N* = 1968; validation cohort: *N* = 842.

Characteristic	Discovery Non-Users (*N* = 1772)	Discovery Statin Users (*N* = 196)	Validation Non-Users (*N* = 759)	Validation Statin Users (*N* = 83)
Age, years, mean (SD)	37.96 (11.50)	54.15 (8.89)	37.45 (11.02)	53.33 (10.24)
BMI, mean (SD)	28.89 (6.05)	31.10 (5.43)	28.64 (5.86)	30.46 (5.43)
LDL, mean (SD)	3.04 (0.85)	2.53 (0.89)	3.03 (0.88)	2.57 (1.11)
HDL, mean (SD)	1.34 (0.37)	1.25 (0.34)	1.36 (0.38)	1.32 (0.39)
Triglycerides, median [IQR]	1.15 [0.80–1.70]	1.53 [1.06–2.00]	1.10 [0.80–1.61]	1.42 [0.99–1.95]
Fasting time, min, median [IQR]	156 [85–554.5]	191 [109.5–637]	150 [88–568]	192 [95–698.5]

## Data Availability

The original contributions presented in this study are included in the article/Supplementary Materials. Further inquiries can be directed to the corresponding author.

## Supplementary Materials

The following supporting information can be downloaded at: https://www.mdpi.com/article/10.3390/ph19091475/s1, Figure S1: F-Statistics of Instrument variables used in the analysis; Figure S2: One sample MR; Table S1: Metabolites associated with Triglycerides; Table S2: Metabolites associated with LDL; Table S3: Metabolites associated with HDL; Table S4: Common metabolites between Triglycerides, HDL, and LDL; Table S5: Details of SNPs used in the One sample MR and MR-G×E; Table S6: R-Square analysis and F-Statistics; Table S7: One sample MR and MR-G×E results; Table S8: MR-G×E results P value FDR corrections.
