# Supplementary material for: Gene–Environment Interaction Mendelian Randomization Reveals Statin-Dependent Associations Between Metabolites and Lipid Traits
Source: Pharmaceuticals (Basel). 2026 Sep 17;19(9):1475. doi: 10.3390/ph19091475 (PMC13609980; doi:10.3390/ph19091475)
Supplement: Supplementary file 1 [file pharmaceuticals-19-01475-s001.zip › pharmaceuticals-4543492-supplementary figures.pdf]

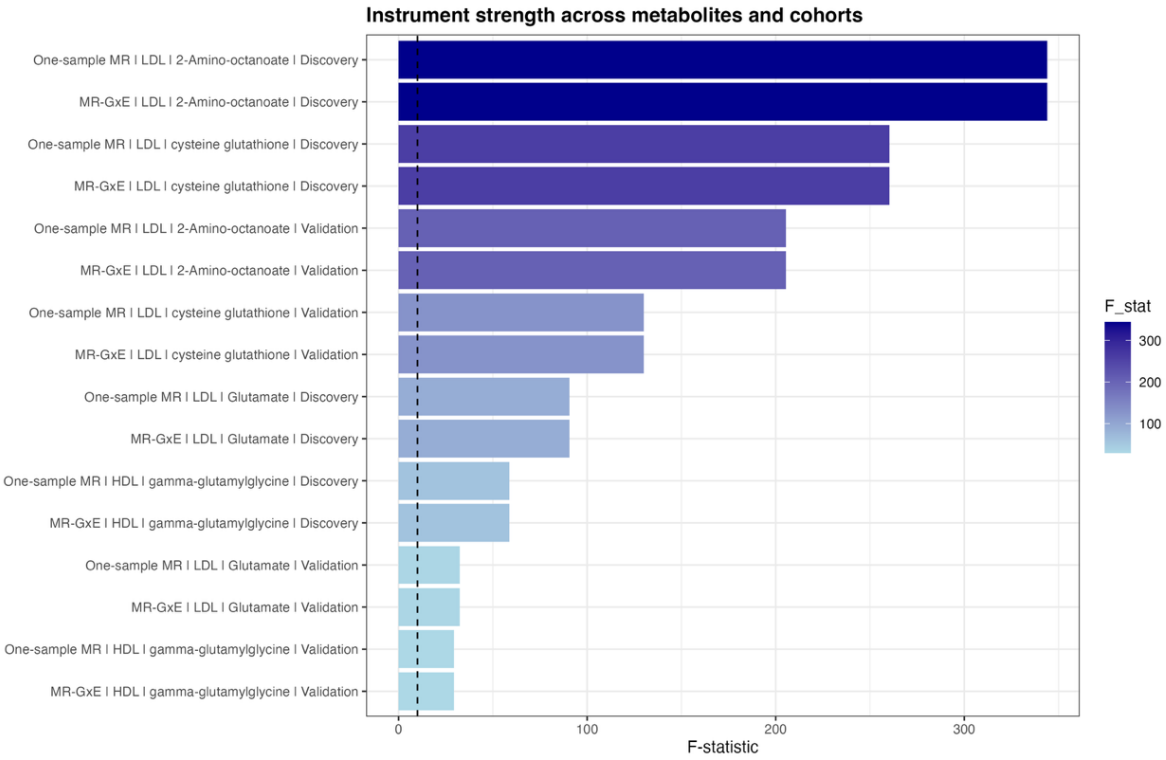

Figure S1: F-Statistics of Instrument variables used in the analysis

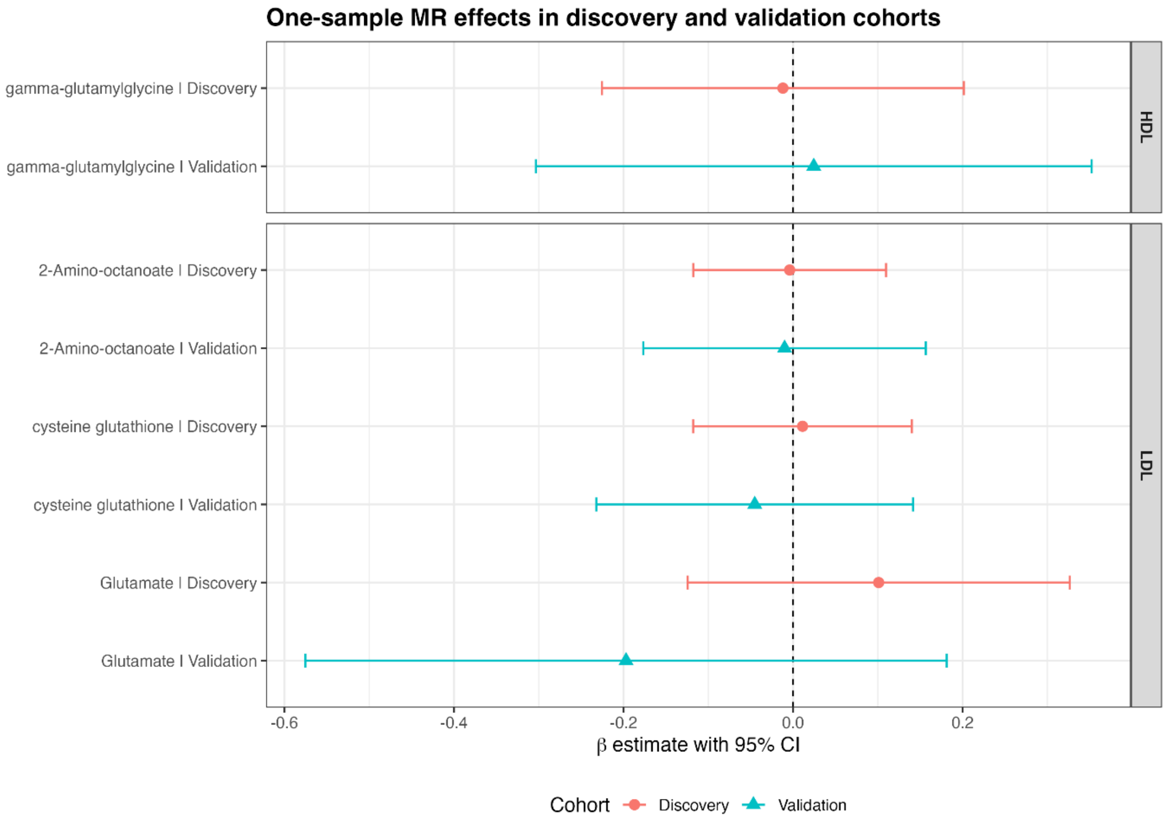

Figure S2: One sample MR: Forest plot represents the β estimates (95% CI) of genetically predicted metabolites on HDL and LDL levels in discovery and validation cohort of overall population. Red point represents the average causal effect in discovery cohort while blue point represents the average causal effect in validation cohort.
